# CRISPRing *EDR1*: Harmonizing grapevine defense and growth dynamics

**DOI:** 10.1093/plphys/kiae181

**Published:** 2024-03-22

**Authors:** Ritu Singh

**Affiliations:** Assistant Features Editor, Plant Physiology, American Society of Plant Biologists; Department of Plant Science, University of California, Davis, CA 95616, USA

Powdery mildew, caused by the fungus *Erysiphe necator*, remains a significant concern for grape yields worldwide, persisting for over a century and posing ongoing challenges for growers. Effective management strategies demand integrated approaches, incorporating cultural practices, strategic pesticide application, and the development of resistant varieties (van Esse et al. 2020). Traditionally, efforts to breed resistant varieties have mainly focused on identifying and integrating specific resistance genes (*R* genes) (Dangl and Jones 2001). However, reliance solely on single *R* genes has proven ineffective over time because pathogens can evolve to overcome them (Dangl et al. 2013) and incorporating multiple *R* genes is time-consuming (Xiao et al. 2019).

Alternatively, researchers have explored exploiting recessive mutations in host susceptibility genes (*S* genes) to confer resistance (van Schie and Takken 2014). Unlike *R* genes, resistance mediated by *S* genes tends to be more durable. Moreover, disrupting a single *S* gene is simpler and faster than integrating multiple *R* genes. *Enhanced disease resistance 1* (*EDR1*) is one such *S* gene identified in an *Arabidopsis* mutant, which showed increased disease resistance to *Golovinomyces cichoracearum*, a causal agent of powdery mildew (Frye and Innes 1998). *EDR1* is highly conserved across plant species and negatively regulates plant defense responses (Shen et al. 2011; Ma et al. 2021).

In this issue of *Plant Physiology*, Yu et al. (2024) identified an ortholog of the *Arabidopsis EDR1* gene in grapevine (*VviEDR1*) through multiple sequence alignment and phylogenetic analysis. Expression analysis in susceptible Thompson Seedless grapevine leaves suggests continual downregulation of *VviEDR1* after *E. necator* inoculation, indicating the role of *VviEDR1* in grapevine powdery mildew infection. To delve deeper, the authors generated *VviEDR1* mutants through CRISPR-Cas9. Most of the obtained *VviEDR1* mutant plants exhibited growth defects and did not survive. The only edited regenerated plants that showed wild-type growth levels were *VviEDR1-chimeric* edited lines, with more than 2 different mutations in a single plant.

To understand the role of *VviEDR1* in grapevine powdery mildew response, the authors infected the leaves of *VviEDR1-chi* and wild-type (WT) plants with *E. necator*. The *VviEDR1-chi* mutant leaves showed more resistance against powdery mildew compared to WT. Furthermore, when *VviEDR1-chi* and WT plants were grown in the greenhouse infected with *E. necator* for over 2 years, *VviEDR1-chi* lines showed enhanced resistance to powdery mildew compared to WT, without compromising growth. This suggests that *VviEDR1-chi* mutants provide resistance against *E. necator* without growth penalty.

To further investigate the mechanism by which *VviEDR1*-*chi* confers resistance to powdery mildew without affecting plant growth and development, the authors analyzed the types and proportions of *VviEDR1* mutations in various tissues of *VviEDR1-chi* lines using next-generation sequencing and high-throughput tracking of mutations platforms. Interestingly, the authors found that in the newly unfolded leaves, the proportion of mutated *VviEDR1* was < 20%, and as leaf growth progressed, the proportion of mutated *VviEDR1* increased, with mature leaves containing > 60% mutated *VviEDR1*. Similar trends were observed in stem tissues, with higher mutation proportions in lower-positioned and older tissues compared to newly developing tissues. Additionally, very few mutations were detected in the terminal and axillary buds (<1.0%), indicating that *VviEDR1* mutations were absent or rare in the shoot apical meristem. Furthermore, approximately 10% of the mutated *VviEDR1* occurred in lateral roots, whereas no mutations were detected in aerial and adventitious roots of *VviEDR1-chi* lines. Overall, the results suggest that a higher ratio of mutated cells in the lower-positioned and older senescent tissues than in the newly developing tissue from the apical meristem. Further, the authors also find consistent mutation regulation and a relatively stable mutation ratio among the different clones, indicating that the chimeric trait of *VviEDR1-chi* could be inherited by clonal propagation.

Because the number of mutations varies at different developmental stages of leaves, the authors evaluated powdery mildew resistance of both young and mature leaves by natural infection in the greenhouse. Whitish mildew colonies were observed on young leaves of both WT and *VviEDR1-chi* lines, whereas mature leaves of *VviEDR1-chi* exhibited strong resistance to powdery mildew with no visible colonies. Transcriptomic analysis of mature leaves of WT and *VviEDR1-chi* lines suggested that *VviEDR1* edited lines regulate resistance to powdery mildew by activating multiple defense pathways, including callose deposition, increased salicylic acid and ethylene production, H_2_O_2_ production, and host cell death.

Overall, the study suggests that *VviEDR1* mutation increases powdery mildew resistance at the expense of severe growth defects. The *VviEDR1* homozygous and bi-allelic edited lines died due to the induction of a severe spontaneous hypersensitivity reaction. In *VviEDR1-chi* plants, almost no mutated *VviEDR1* existed in the apical meristem, so the cells of the shoot apical meristem could divide and differentiate normally to maintain plant growth **(**Fig. 1**)**. While the *VviEDR1* mutation ratio increased with increasing leaf age, abundant mutations accumulated in mature leaves, conferring leaf resistance to powdery mildew by hypersensitive response-like cell death and H_2_O_2_ accumulation in the impacted epidermal cells and neighboring cells. Thus, the *VviEDR1-chi* maintains the balance between growth and defense.

**Figure 1.**
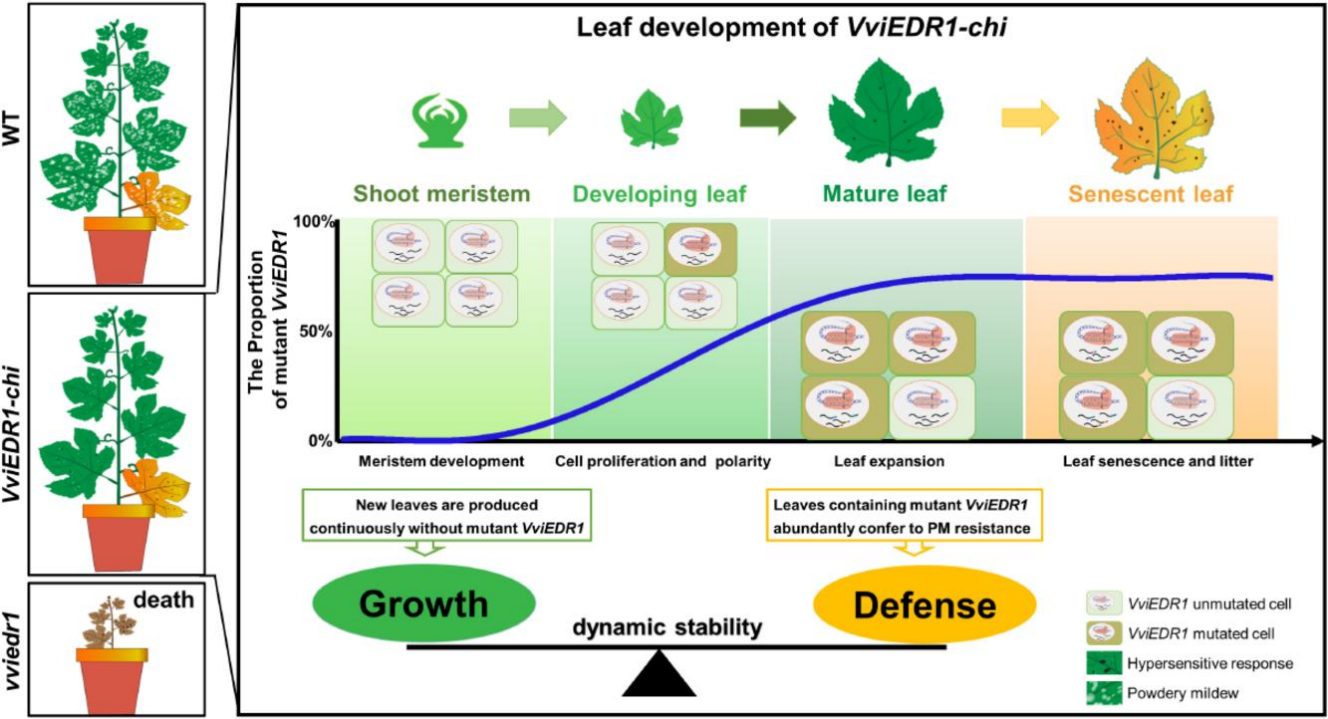
Schematic model depicting how *VviEDR1-chi* lines enhance resistance to powdery mildew without compromising growth (Figure 8 of Yu et al. 2024). The *VviEDR1-chi* lines express both the wild-type *VviEDR1* and mutant *Vviedr1* alleles as chimera (*VviEDR1-chi*), ensuring the dynamic stability of the *VviEDR1* mutation. Almost no *VviEDR1* mutation is detected in the apical meristem, allowing cells of the shoot apical meristem to divide and differentiate normally for plant growth. As leaves mature, the *VviEDR1* mutation ratio increases, resulting in abundant accumulation of mutation in mature leaves and conferring resistance against powdery mildew.

## Data Availability

No data is generated in this study.
